# An Evaluation of Referrals and Attendance at a Perinatal Specialist Mental Health Service

**DOI:** 10.1192/bjo.2024.463

**Published:** 2024-08-01

**Authors:** Francesca Best, Mennatullah Dakroury

**Affiliations:** 1 Newcastle University, Newcastle, United Kingdom; 2 Cumbria, Northumberland, Tyne and Wear NHS Foundation Trust, Newcastle, United Kingdom

## Abstract

**Aims:**

This service evaluation had four aims:

1. Breakdown the sources of referrals to one Perinatal Specialist Mental Health Service.
2. Calculate the average waiting time from referral to an initial assessment.
3. Analyse the Did Not Attend (DNA) rate for initial assessments.
4. Suggest possible service improvements to reduce waiting times and DNA rates.

**Methods:**

Referrals made in the period May−July 2023 to the Cumbria, Northumberland, Tyne and Wear (CNTW) Perinatal Specialist Mental Health Service were collated. Data regarding the source of referral, demographic details of the patient, whether they were accepted for assessment and whether they did or did not attend their assessment and the outcome of the case was analysed.

**Results:**

Midwives and GPs made the greatest number of referrals (37% and 26% respectively). Out of 263 referrals, 47 did not meet the criteria for an initial assessment – the largest single contributor to this number being referrals from GPs. Just under 16% of referrals made by GPs were found more suited to primary care services after initial assessment compared with 11% amongst referrals from midwives.

The average waiting time from an accepted referral to assessment was 29.85 days. This is higher than the CNTW two-week wait target.

Of the 203 patients offered assessments, there were 20 occasions on which patients DNA. Those who DNA were more likely to have history of domestic abuse (55% compared with 48% amongst those who attended their assessment first-time). Of the patients who DNA their first appointment, 1/3 attended future appointments.

Text reminders about appointments proved extremely popular; where there was information available, 98% of patients were agreeable to text reminders about their appointments.

**Conclusion:**

Waiting times could be reduced by implementing tighter guidelines for referrals and further educating referrers on the specific role of the perinatal service in contrast to primary psychological services, thus reducing unnecessary assessments.

Text reminders should continue to be used in addition to offering assessments at home where suitable. In several cases, patients who had forgotten about their appointment were still agreeable to assessment when met at home.

Future research could be carried out in collaboration with patients who DNA to better understand the barriers they face to attendance.

